# Radiation Augments the Local Anti-Tumor Effect of *In Situ* Vaccine With CpG-Oligodeoxynucleotides and Anti-OX40 in Immunologically Cold Tumor Models

**DOI:** 10.3389/fimmu.2021.763888

**Published:** 2021-11-15

**Authors:** Alexander A. Pieper, Luke M. Zangl, Dan V. Speigelman, Arika S. Feils, Anna Hoefges, Justin C. Jagodinsky, Mildred A. Felder, Noah W. Tsarovsky, Ian S. Arthur, Ryan J. Brown, Jen Birstler, Trang Le, Peter M. Carlson, Amber M. Bates, Jacquelyn A. Hank, Alexander L. Rakhmilevich, Amy K. Erbe, Paul M. Sondel, Ravi B. Patel, Zachary S. Morris

**Affiliations:** ^1^Department of Human Oncology, University of Wisconsin School of Medicine and Public Health, Madison, WI, United States; ^2^Department of Biostatistics and Medical Informatics, University of Wisconsin School of Medicine and Public Health, Madison, WI, United States; ^3^Department of Pediatrics, University of Wisconsin School of Medicine and Public Health, Madison, WI, United States; ^4^Department of Radiation Oncology and Bioengineering, University of Pittsburgh Hillman Cancer Center, Pittsburgh, PA, United States

**Keywords:** *In situ* vaccine, cold tumor models, OX40 agonist, CpG – oligonucleotides, radiation therapy, radioimmunotherapy

## Abstract

**Introduction:**

Combining CpG oligodeoxynucleotides with anti-OX40 agonist antibody (CpG+OX40) is able to generate an effective *in situ* vaccine in some tumor models, including the A20 lymphoma model. Immunologically “cold” tumors, which are typically less responsive to immunotherapy, are characterized by few tumor infiltrating lymphocytes (TILs), low mutation burden, and limited neoantigen expression. Radiation therapy (RT) can change the tumor microenvironment (TME) of an immunologically “cold” tumor. This study investigated the effect of combining RT with the *in situ* vaccine CpG+OX40 in immunologically “cold” tumor models.

**Methods:**

Mice bearing flank tumors (A20 lymphoma, B78 melanoma or 4T1 breast cancer) were treated with combinations of local RT, CpG, and/or OX40, and response to treatment was monitored. Flow cytometry and quantitative polymerase chain reaction (qPCR) experiments were conducted to study differences in the TME, secondary lymphoid organs, and immune activation after treatment.

**Results:**

An *in situ* vaccine regimen of CpG+OX40, which was effective in the A20 model, did not significantly improve tumor response or survival in the “cold” B78 and 4T1 models, as tested here. In both models, treatment with RT prior to CpG+OX40 enabled a local response to this *in situ* vaccine, significantly improving the anti-tumor response and survival compared to RT alone or CpG+OX40 alone. RT increased OX40 expression on tumor infiltrating CD4+ non-regulatory T cells. RT+CpG+OX40 increased the ratio of tumor-infiltrating effector T cells to T regulatory cells and significantly increased CD4+ and CD8+ T cell activation in the tumor draining lymph node (TDLN) and spleen.

**Conclusion:**

RT significantly improves the local anti-tumor effect of the *in situ* vaccine CpG+OX40 in immunologically “cold”, solid, murine tumor models where RT or CpG+OX40 alone fail to stimulate tumor regression.

## Introduction

Immunotherapy is helping to improve survival and cure rates for many different types of cancer; however, most patients receiving immunotherapy still show no response to treatment (1, 2). The tumor microenvironment (TME) can influence a patient’s response to immunotherapy (3). In general, immunologically “hot” tumors respond better to immunotherapy than immunologically “cold” tumors. An immunologically “hot” tumor is one in which the TME typically demonstrates high levels of infiltrating T cells, proinflammatory cytokines, PD-L1 expression, and tumor cells with elevated mutation burden and expression of actionable neoantigens (4, 5). An immunologically “cold” tumor is one that typically lacks most or all of these characteristics.

*In situ* vaccination is a treatment strategy that aims to modify the TME in a manner that enables T cell recognition of tumor antigens not recognized prior to that therapy, leading to a more diversified adaptive anti-tumor immune response (6). In contrast to traditional vaccinations, which often deliver a known antigen to activate immunity, *in situ* vaccination does not depend on the identification or exogenous delivery of a tumor antigen. *In situ* vaccination instead seeks to destroy or modify tumor cells and/or the TME in a way that overcomes barriers to tumor antigen presentation to activate adaptive anti-tumor immunity. This is commonly pursued using treatments that increase tumor immunogenicity, increase tumor infiltration by immune cells, and increase immune cell activation (7, 8). *In situ* vaccination efforts are particularly appealing clinically because they are commonly able to make use of off-the-shelf treatments to achieve an exquisitely personalized anti-tumor immune response by activating a patient’s own immune system to recognize potentially any of the unique antigens present in that patient’s cancer. By enabling a systemic adaptive immune response against a large repertoire of tumor antigens, *in situ* vaccination approaches may limit the potential for therapeutic resistance due to tumor heterogeneity or antigen loss (7).

In prior studies, combining CpG and agonistic anti-OX40 antibody (OX40) was able to activate an *in situ* vaccine effect and generate substantial preclinical anti-tumor activity in some tumor models, including the A20 lymphoma model (9–11). CpG oligodeoxynucleotides, synthetic analogs resembling unmethylated bacterial DNA, are pathogen-associated molecular pattern (PAMP) molecules that bind and activate toll-like receptor 9 (TLR9) expressed within the endosomes of immune cells including macrophages, dendritic cells, and other antigen presenting cells (12). TLR9 activation results in NF-κB activation, production of Type I interferons (IFNs), and increased T cell priming. OX40 receptor is a T cell co-stimulatory receptor, transiently expressed by CD4+ and CD8+ T cells following T cell receptor/CD3 crosslinking, and it is constitutively expressed by mouse CD4+ T regulatory cells (Tregs) (13). Binding of OX40 receptor on CD8+ or CD4+ non-regulatory T cells promotes their expansion and survival (14–16). OX40 receptor engagement on Tregs has been shown to decrease IL-10 production, downregulate FoxP3 expression, and result in Treg cell death (13, 17, 18). Preclinical studies have demonstrated that IT injections of CpG and OX40 work synergistically to cure mice of local and distant untreated tumors in a T cell dependent manner (11). This preclinical anti-tumor activity led to the initiation of a clinical trial, which is currently ongoing (NCT03831295), where this *in situ* vaccine is being tested in patients with advanced or metastatic solid tumors.

We previously reported on a separate *in situ* vaccine approach that is effective against small syngeneic tumors that express the disialoganglioside GD2. This approach involved direct intratumoral (IT) injections of an immunocytokine (IC), a GD2-specific monoclonal antibody (mAb) genetically linked to interleukin-2 (IL-2) (19–22). The antibody portion of the IC binds to GD2 on the surface of certain tumor cells and induces antibody dependent cell-mediated cytotoxicity (ADCC) *via* Fc γ receptor interactions (21). With the IC bound to tumor cells, IL-2 is concentrated in the TME and stimulates NK and T cell expansion (21). However, in well-established tumors that have undergone more immunoediting, or in immunologically “cold” tumors with few tumor infiltrating lymphocytes (TILs), we observed that single agent treatment with IT-IC was ineffective and did not activate either an *in situ* vaccine effect or anti-tumor response (23). In these settings of relatively immunosuppressive TMEs, local radiation therapy (RT) could be used in conjunction with IT-IC to restore the *in situ* vaccine effect of this locally injected immunotherapy (23). Prior studies from our group and others had demonstrated that RT was capable of locally transforming an immunologically “cold” TME to one that is phenotypically “hot” through local activation of a type I interferon response *via* the cGAS/STING pathway, increased infiltration and activation of lymphocytes and dendritic cells in the radiated TME, upregulation of the major histocompatibility complex-I (MHC-I) and FAS expression on tumor cells, and the increased tumor expression of neoantigens (6, 24–30). In the immunologically “cold” B78 murine melanoma model, we observed that a combined modality *in situ* vaccine regimen using RT+IT-IC together with immune checkpoint blockade cured the majority of treated-mice bearing relatively large primary tumors and disseminated metastases by inducing a T cell-mediated anti-tumor response (23). This regimen is currently under clinical investigation in a Phase I trial enrolling patients with metastatic melanoma (NCT03958383).

Our experience with the IT-IC *in situ* vaccine regimen led us to test whether an unrelated CpG+OX40 *in situ* vaccine would be similarly ineffective as IT-IC when administered to immunologically “cold” tumor models, and whether RT could enable a response to this regimen in these models. We demonstrate that CpG+OX40 is insufficient to consistently cause tumor regression in two immunologically “cold” tumor models, despite its potent efficacy in the A20 lymphoma model. We also found that treating immunologically “cold” tumors with a single fraction of RT prior to injecting the CpG+OX40 *in situ* vaccine regimen markedly improved the local anti-tumor response. The combination of RT+CpG+OX40 resulted in favorable improvements in the ratio of effector T cells to Tregs in the TME, increased expression of proinflammatory genes in the TME, and activated CD4+ and CD8+ T cells in the tumor draining lymph node (TDLN) and spleen.

## Methods

### Mice

Female C57BL/6 and BALB/c mice were purchased from Taconic Farms (TAC, Germantown, NY). Mice were 7-8 weeks old when purchased and were housed in accordance with the Guide for Care and Use of Laboratory Mice. Experiments were performed under an animal protocol approved by the institutional animal care and use committee.

### Cell Culture

A20 lymphoma was obtained from Stephen Gillies PhD (Carlisle MA) in 2017. In order to get consistent tumor engraftment, A20 cells were harvested from a growing *in vivo* flank tumor, digested into a single cell suspension, and passaged *in vivo* in the peritoneal cavity of a naïve BALB/c mouse, prior to maintaining them *via in vitro* culture. B78-D14 (B78) melanoma was derived from B16 melanoma and was obtained from Ralph Reisfeld PhD (La Jolla CA) in 2002. 4T1 triple negative breast cancer cell line was obtained from ATCC in 2018. B78 and 4T1 cells were grown in RPMI-1640 (Corning) supplemented with 10% heat-inactivated FBS (Gibco), 2 mmol/L *L*-Glutamine (Corning), 100 U/mL penicillin (Corning), and 100 µg/mL streptomycin (Corning). A20 cells were grown in the same conditions as B78 and 4T1 with the addition of 50 mM 2-Mercaptoethanol. Mycoplasma testing *via* qPCR was routinely done.

### *In Vivo* Tumor Models

A20 (5x10^6^), B78 (2x10^6^), and 4T1 (2x10^5^) cells were injected intradermally (rather than subcutaneously) into the right flank of C57BL/6 (B78) or BALB/c (A20, 4T1) mice (31). Tumor volumes were measured and calculated as previously described (23). Once average tumor volumes reached target size, mice were randomized into their treatment groups so that each group had a similar average starting tumor volume. Mice were euthanized when the longest tumor dimension reached 20 mm or the mouse became moribund due to metastatic disease.

### Radiation

*In vivo* local RT was dosed to flank tumors using an X-Rad 320 irradiator (Precision X-Ray, Inc.). Mice were immobilized using custom made lead jigs that only exposed the mouse’s dorsal right flank to the radiation field. RT was delivered in one fraction totaling either 8 Gy (4T1) or 12 Gy (B78), as previously described (23, 32). In the experiments where mice received RT, the day of radiation was defined as day 0 of the experiment.

### Immunotherapy and Antibodies

CpG 1826 was purchased from Integrated DNA Technologies. OX40 antibody [Anti-OX40 (CD134) antibody, rat immunoglobulin G1, OX86 clone, European Collection of Cell Cultures] was harvested and isolated from the ascites of immunodeficient mice, as previously described (33). CpG (50 µg) and/or OX40 (4 µg, 20 µg, or 100 µg) were injected IT with a 29 ½ gauge insulin syringe in 60 µL PBS every other day for three total doses (days 0, 2, 4 or days 5, 7, 9 depending on the experiment).

The following antibodies were used for flow cytometry analysis: anti-CD16/32 (93), CD45 BV510 (30-F11), CD45 FITC (30-F11), CD3 PE-Cy5 (145-2C11), CD4 BV785 (GK1.5), CD19 PE-Cy5 (6D5), CD19 APC (6D5), CD19 BV421 (6D5), Ly6G Alexa647 (1A8), IFNγ PE-Cy7 (XMG1.2), and OX40 PE (OX-86) all from BioLegend; CD8 APC-R700 (53-6.7), CD25 BB515 (PC61), NK1.1 PE-CF594 (PK136), Ly6C BV605 (AL-21), and CD11b V450 (M1/70) all from BD Biosciences; FoxP3/Transcription Factor Staining Buffer Set and FoxP3 PE-Cy7 (FJK-16s) from eBioscience. GhostRed780 Viability Dye (Tonbo Biosciences) was used for live/dead staining.

### OX40 Expression Following IT CpG

A20 or B78 tumors were implanted as described above. A20 or B78 tumor bearing mice were randomized into 2 treatment groups (n=4-6 per group) and injected IT with PBS or CpG (50µg). After treatment, mice were euthanized with CO_2_ and tumors excised. A20 tumors were disaggregated manually using the plunger of a syringe and a 70 µm filter. B78 tumors were disaggregated in 2.5 mL complete RPMI, 2.5 mg of collagenase type IV and 250 µg of DNAase using a Miltenyi gentleMACS Octo Dissociator and passed through a 70 µm filter. Three million cells from each individual tumor sample (A20 or B78) were added to individual wells of a 96-well round bottom plate. Leftover cells were pooled and used for fluorescence minus one controls; for live/dead controls, 1.5 million cells were killed *via* heat shock and mixed with 1.5 million live cells. Samples were stained with GhostRed780 Viability Dye for 30 minutes at 4°C, then incubated with anti-CD16/32 for 10 minutes at room temperature to reduce nonspecific binding. Each sample was stained for 30 minutes at 4°C with antibodies in brilliant stain buffer (BD Biosciences). Samples were fixed and permeabilized overnight at 4°C with the FoxP3/Transcription Factor Staining Buffer Set following kit instructions. Samples were stained with FoxP3 PE-Cy7 for 30 minutes at 4°C.

All data were collected on an Attune flow cytometer (ThermoFisher) and analyzed with FlowJo v10 software (BD). The gating strategy used to quantify the data for various figures are referenced in figure legends. CD4+ Tregs were defined as CD45+ CD3+ CD4+ CD25+ FoxP3+. The CD4+ T cells that were not double positive for CD25+ and FoxP3+ were defined as non-Treg CD4+ cells.

### Tumor Infiltrating Immune Cell Analysis

B78 tumors were implanted as described above. Mice were randomized into 4 treatment groups and received treatment as described above (n=5 per group): PBS, CpG+OX40, RT, RT+CpG+OX40. Tumors were harvested, digested, and stained as described above with the exception that tumor cell contents were added to flow tubes rather than a 96 well plate. Cell staining, data collection, and data analysis were conducted as described above. The gating strategy used to quantify the data for various figures are referenced in figure legends. CD4+ Tregs and non-Treg CD4+ cells were defined as described above.

### Gene Expression

For tumor gene expression analyses, freshly dissected specimens were homogenized using a Bead Mill Homogenizer (Bead Ruptor Elite, Omni International Cat # 19- 040E). Total RNA was extracted using RNeasy Mini Kit (Qiagen, Germany, Cat # 74106) according to the manufacturer’s instructions. cDNA was derived using QuantiTect Reverse Transcription Kit (Qiagen, Germany, Cat # 205314) according to the manufacturer’s instructions. Quantitative polymerase chain reaction (qPCR) was performed using PowerUp SYBR Green qPCR Master Mix. The reaction (5µL total volume) was prepared using Labcyte Echo 550 and MANTIS liquid handling systems. Thermal cycling conditions (QuantStudio™ 6, Applied Biosystems) included the UDG activation stage at 50°C for 2 min, followed by Dual-Lock™ DNA polymerase activation stage at 95°C for 2 min followed by 40 cycles of each qPCR step: denaturation at 95°C for 15s and annealing/extension at 60°C for 1 min. A melt curve analysis was done to ensure specificity of the corresponding qPCR reactions. For data analysis, the cycle threshold (Ct) values were exported to an Excel file and fold change was calculated using the ΔΔCt method. ΔΔCt values were then imported into Prism. *Hprt*, *Pgk1*, and *Tbp* were used as endogenous controls.

### T Cell Activation

B78 tumors were implanted, grown, and mice were randomized and treated as described above (n=4-5 per group) with: PBS, CpG+OX40, RT, RT+CpG+OX40. Tumor draining lymph nodes (TDLNs) and spleens were harvested on day 12 after RT and digested using the plunger of a syringe and a 70 µm filter. Red blood cell lysis was performed on spleens with ACK lysis buffer (Invitrogen). 3 million cells were added to a 96 well plate for flow cytometry analysis. Samples were stained for viability, Fc blocked, and surface stained as previously described. Samples were fixed with 2% paraformaldehyde (PFA) for 20 minutes at room temperature, left covered in aluminum foil at 4°C overnight, permeabilized the following day with 1X perm buffer (eBioscience) for 20 minutes at room temperature, and then incubated with anti-IFNγ antibody. Data were collected and analyzed as previously described above. Until the samples were fixed in PFA, secondary lymphoid organs and samples were kept in media or flow buffer containing 1X protein transport inhibitor (PTI) (eBioscience, Cat # 00-4980-03).

### Statistical Analysis

Average group tumor volumes are plotted showing mean +/- standard error of the mean (SEM). Tumor volume plots were summarized by time-weighted average (area under the volume-time curve, calculated using trapezoidal method). Time-weighted averages were compared between treatment groups overall by Kruskal-Wallis tests. If significance was found using Kruskal-Wallis test, then pairwise comparisons were conducted using Mann-Whitney tests. Survival data were plotted using Kaplan-Meier methods and analyzed using log-rank comparisons. Despite the large number of tests, no p-value correction methods were used to account for inflated type 1 error.

Flow cytometry results are plotted as mean +/- standard error of the mean. The following steps were taken to quantify the flow results showing fold change in median fluorescent intensity (MFI): For each separate experiment, the group of mice treated with PBS was used to calculate a PBS MFI average. Each mouse’s MFI value was then divided by the intra-experimental PBS MFI average and reported as a fold change. Experimental fold change differences were grouped based on treatment and analyzed using a Mann-Whitney test. Flow results in figures were analyzed using a Mann-Whitney test or a one-way ANOVA with Tukey’s multiple comparison test (as clarified in figure legends). Gene expression changes were analyzed using Kruskal-Wallis test followed by Dunn pairwise comparison test with Holm adjustment for p-values. P values < 0.05 were considered significant and were indicated in figures as follows: *, P < 0.05; ** P < 0.01; *** P < 0.001; **** P < 0.0001; NS – nonsignificant.

## Results

### CpG+OX40 *In Situ* Vaccine Cures Mice of A20 Lymphoma but Not B78 Melanoma

**Figure 1:**
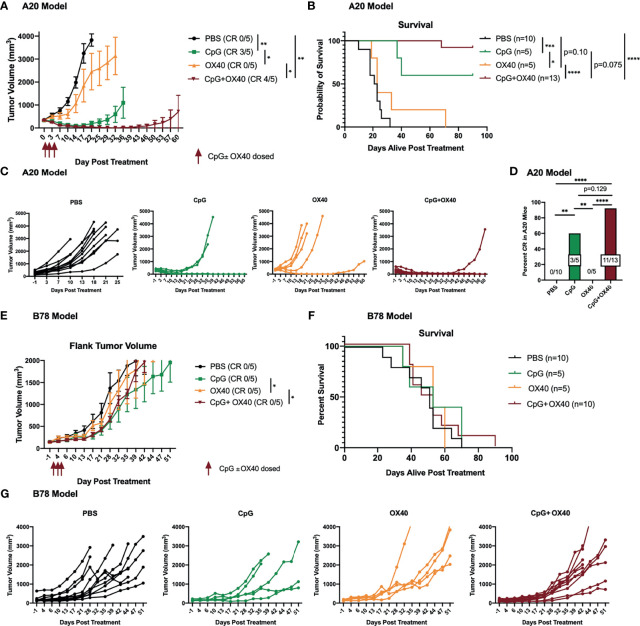
CpG+OX40 *In Situ* Vaccine Cures Mice of A20 Lymphoma but Fails to Cause Tumor Regression in B78 Melanoma. **(A)** Average tumor volume plots (+/- standard error of the mean) from one representative experiment, and **(B)** combined overall survival in the A20 model showing group responses to PBS (black), CpG (green), OX40 (yellow), and CpG+OX40 (red). The number of mice that were cured of their tumor burden in **(A)** are shown in parentheses (CR). The anti-tumor response of CpG alone and OX40 alone were tested in one experiment (in **A**), while the strong anti-tumor response of CpG+OX40 in the A20 model was tested in two independent experiments [shown together in **(B)**]. **(C)** Combined individual animal tumor growth plots for each animal shown in **(A, B)**. **(D)** The percent of A20 tumor bearing mice in **B** that were cured of their tumor burden following treatment with PBS, CpG, OX40, and CpG+OX40. **(E)** Tumor volume plots (+/- standard error of the mean) from one representative experiment, and **(F)** overall survival in the B78 model showing group responses to PBS (black), CpG (green), OX40 (yellow), and CpG+OX40 (red). The number of mice that were cured of their tumor burden in **E** are shown in parentheses. The anti-tumor response of CpG alone and OX40 alone were tested in one experiment in **(E)**, while the lack of anti-tumor response of CpG+OX40 in the B78 model was tested in two independent experiments [shown together in **(F)**]. **(G)** Individual tumor growth curves of mice in **E** showing responses to PBS (black), CpG (green), OX40 (yellow), and CpG+OX40 (red) in the B78 model. Red arrows shown in **(A, E)** indicate the days CpG and/or OX40 were dosed IT (d. 0, 2, 4). In both tumor models, n=5-8 per group per experiment. P values for average tumor volume plots calculated using time-weighted average analysis. P values for overall survival calculated *via* log rank test. P values for CR rates calculated *via* one-way ANOVA. *P ≤ 0.05; **P ≤ 0.01; ***P ≤ 0.001; ****P ≤ 0.0001.

Initially, we confirmed that when we administered the *in situ* vaccine regimen of CpG+OX40, we observed efficacy that was similar to that previously reported (9–11). For this, mice bearing flank A20 tumors (~350 mm^3^) were randomized into 4 treatment groups: PBS, CpG, OX40, or CpG+OX40. OX40 alone did not significantly improve the local tumor response or overall survival compared to the control (**Figures 1A, B**). CpG alone significantly improved tumor control and survival compared to PBS and OX40 (**Figures 1A–C**). The *in situ* vaccine CpG+OX40 generated the strongest anti-tumor response, significantly slowing tumor progression, improving survival compared to PBS and OX40, and curing significantly more mice than PBS and OX40 (**Figures 1A–D**).

Because of the potent anti-tumor response elicited by CpG+OX40 in the A20 model, we sought to investigate its anti-tumor potential in the immunologically “cold” B78 melanoma model (32, 34). Mice bearing B78 flank tumors (~150 mm^3^) were randomized and treated with PBS, CpG, OX40, or CpG+OX40. In the B78 model, CpG+OX40 did not significantly improve tumor control or overall survival compared to PBS (**Figures 1E–G**). In fact, there was no difference in survival between any of the cohorts. Recognizing there are immunologic differences between the A20 and B78 models, we tested whether additional T cell activation, in the form of additional agonist OX40 antibody, would stimulate an anti-tumor response. We conducted an OX40 dose escalation study where mice bearing B78 tumors were treated with CpG (50 µg) and various doses of OX40 (4 µg, 20 µg, or 100 µg). Additional OX40 stimulation, in the form of increased doses of OX40 antibody, did not improve the anti-tumor response or survival (**Supplementary Figure 1**).

### CpG Increases OX40 Expression on Tumor Infiltrating T Cells in the A20 Model but Not the B78 Model

**Figure 2:**
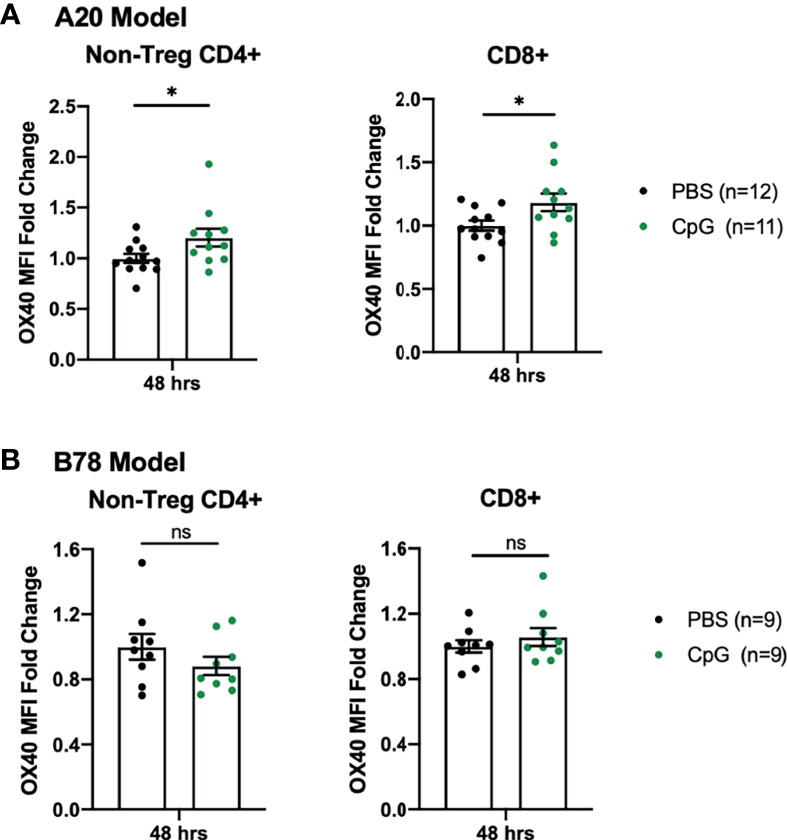
B78 and A20 Tumor Models Demonstrate Differences in Response to CpG Treatment. **(A)** OX40 MFI fold change expression on non-Treg CD4+ or CD8+ tumor infiltrating T cells in the A20 model 48 hours after treatment with PBS (black) or CpG (green). **(B)** OX40 MFI fold change expression on non-Treg CD4+ or CD8+ tumor infiltrating T cells in the B78 model 48 hours after treatment with PBS (black) or CpG (green). The data plotted here are the results from two independent experiments (n=4-6 per group, per experiment). Average OX40 MFI values in the PBS cohort were calculated for each separate experiment, then used to determine fold-change differences for each sample from that particular experiment. Each symbol represents the MFI fold change from one mouse. Flow gating strategy is presented in **Supplementary Figure 3**. P values were calculated using Mann-Whitney tests. *P ≤ 0.05; NS, nonsignificant.

After failing to see tumor regression with CpG+OX40 in the B78 model, we investigated the activity of CpG in the A20 and B78 tumor models. Others have shown the synergistic anti-tumor effect of CpG+OX40 is due, in part, to CpG’s ability to increase OX40 expression on tumor infiltrating CD4+ T helper cells (11). We harvested A20 and B78 tumors 48 hours after IT injections with PBS or CpG and analyzed TILs for OX40 expression. In the A20 model, CpG significantly increased the OX40 MFI on tumor infiltrating non-Treg CD4+ and CD8+ T cells, as reported (11) (**Figure 2A**). In contrast, in the B78 model, treatment with CpG did not increase OX40 expression on the non-Treg CD4+ or CD8+ T cell populations (**Figure 2B**).

### RT Enhances Local Anti-Tumor Response of CpG+OX40 *In Situ* Vaccine in the B78 Model

**Figure 3:**
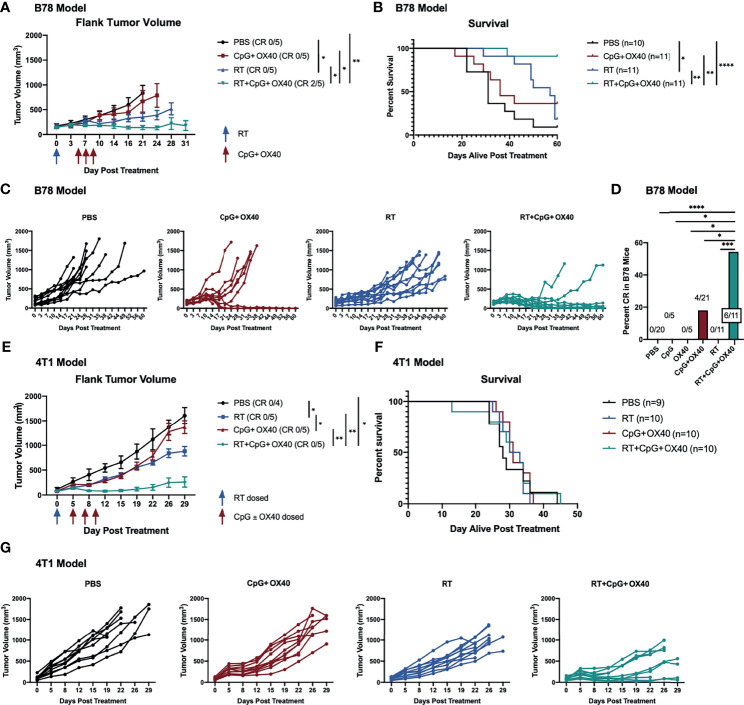
RT Enhances the Local Anti-Tumor Effect of CpG+OX40 in Multiple Tumor Models. **(A)** Average tumor volume plots (+/- standard error of the mean) from a representative experiment, and **(B)** combined overall survival from two independent experiments in the B78 model showing group responses to PBS (black), CpG+OX40 (red), RT (blue), and RT+CpG+OX40 (teal) along with the number of mice that demonstrated a complete response (CR) to treatment. **(C)** Combined individual tumor volume plots from two independent experiments showing each mouse’s response to various treatments shown in **(B)**. **(D)** Number of B78 tumor-bearing mice showing a complete response for the summary of 4 experiments (shown in **Figure 1F** and **Figure 2B**) **(E)** Average tumor volume plots (+/- standard error of the mean) from a representative experiment, and **(F)** combined overall survival from two independent experiments in the 4T1 model showing group responses to PBS (black), CpG+OX40 (red), RT (blue), and RT+CpG+OX40 (teal) along with the number of mice that demonstrated a complete response to treatment. **(G)** Combined individual tumor volume plots from two independent experiments showing each mouse’s response to various treatments for the mice shown in **(E)** Red arrows shown in **(A, E)** indicate the days CpG and/or OX40 were dosed IT (d. 5, 7, 9), while blue arrows shown in **(A, E)** indicate if/when RT was dosed (d. 0). In both models, n=4-6 per group per experiment. P values for average tumor volume plots calculated using time-weighted average analysis. P values for overall survival calculated *via* log rank test. *P ≤ 0.05; **P ≤ 0.01; ***P ≤ 0.001; ****P ≤ 0.0001.

We and others have observed a critical role for RT in augmenting the response to an IT administered *in situ* vaccine regimen (6, 23, 35). Among other anti-tumor mechanisms, RT transiently reduces the tumor Treg population (35), modifying the TME for immune activation with an *in situ* vaccine. At baseline levels, we found that B78 tumors demonstrated significantly increased tumor Treg frequency compared to A20 tumors (**Supplementary Figure 2**). We hypothesized that RT would modulate the immunologically “cold” B78 TME in a manner that would enable response to CpG+OX40. Mice bearing B78 tumors were randomized and treated with PBS, CpG+OX40, RT, or RT+CpG+OX40. RT significantly slowed tumor growth and improved survival compared to PBS (**Figures 3A, B**). However, RT did not cause tumor regression in any mice, and all RT treated mice died of progressive tumor growth (**Figures 3B, C**). CpG+OX40 again failed to significantly slow tumor growth or improve survival compared to PBS (**Figures 3A, B**). Delivering one fraction of 12 Gy RT prior to injections with the CpG+OX40 *in situ* vaccine significantly improved tumor response and animal survival compared to all other groups (**Figures 3A, B**). RT+CpG+OX40 resulted in a complete response in the majority of treated mice (55%) (**Figure 3D**), curing significantly more mice than all other treatments tested in the B78 model.

### RT Enhances Local Anti-Tumor Response of CpG+OX40 *In Situ* Vaccine in the 4T1 Model

To evaluate the generalizability of our observations, we used a second immunologically “cold” model in a distinct strain of mice. Using BALB/c mice bearing 4T1 triple-negative breast cancer flank tumors, we tested whether CpG+OX40 might activate an anti-tumor response and whether this response would be enhanced by combination with RT. 4T1 tumor-bearing mice were randomized and treated with PBS, CpG+OX40, RT, or RT+CpG+OX40. IT injections with CpG+OX40 initially slowed local tumor progression. However, all tumors resumed their rapid growth rate by day 12-15, resulting in no statistical difference in local tumor growth between PBS control and CpG+OX40 treatment groups (**Figures 3E, G**). RT alone significantly slowed tumor growth compared to PBS and CpG+OX40. Combining RT with CpG+OX40 was the only treatment regimen that caused tumor regression in several mice, resulting in significantly improved local tumor response compared to PBS, CpG+OX40, or RT alone (**Figures 3E, G**). Even with this superior local control, however, RT+CpG+OX40 failed to provide a survival benefit in the spontaneously metastatic 4T1 model (**Figure 3F**). Mice from each treatment cohort required euthanasia around the same time (days 25-45) because of the symptomatic progression of spontaneous lung metastases. This may suggest that in immunologically “cold” metastatic tumor settings, local RT to the *in situ* vaccine site plays a critical role in permitting an anti-tumor immune response, but that propagation of this response is poor at distant metastatic tumor sites that are also immunologically cold but not radiated.

### RT+CpG+OX40 Modifies Gene Expression in the B78 TME

**Figure 4:**
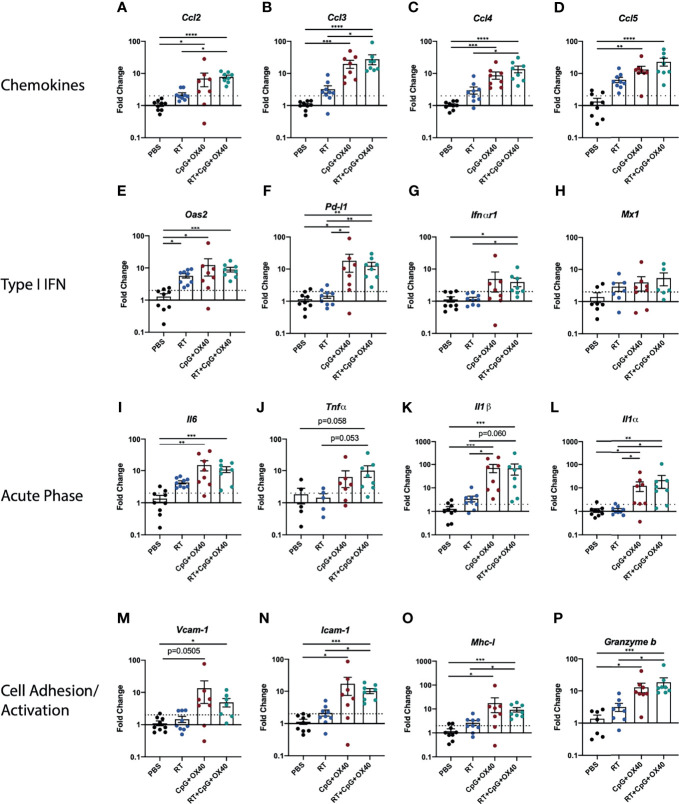
RT+CpG+OX40 Modifies Gene Expression in TME for Favorable Anti-Tumor Response. Fold-change gene expression of **(A–D)** chemokines, **(E–H)** type I IFN pathway genes, **(I–L)** acute phase inflammatory cytokines, and **(M–P)** cell adhesion and immune activation genes in the B78 model on day 14 following treatment initiation with PBS (black), RT (blue), CpG+OX40 (red), and RT+CpG+OX40 (teal). Results are presented from a single set of qPCR analyses that were run simultaneously on tumor tissue samples collected from two independent experiments (n=7-10 total per group). For each gene of interest, significance was determined by Kruskal-Wallis testing; if significance was found groups were compared *via* a Dunn test and adjusted using the Holm method. *P ≤ 0.05; **P ≤ 0.01; ***P ≤ 0.001; ****P ≤ 0.0001.

We next sought to better understand what effects of RT would correlate with its role in enabling local responsiveness to CpG+OX40. RT has been shown to increase TIL frequency through type-I IFN induction, resulting in the production of cytokines, chemokines, and cell adhesion molecules attracting TILs to the radiated tumor site (24–26, 36). RT can also modify tumor cell expression, making irradiated tumor cells more susceptible to immune mediated killing (24). To begin investigating how RT was enabling anti-tumor responses to CpG+OX40, we measured gene expression changes within the TME of B78 tumors 14 days after treatment when group tumor volumes began to show differences between mice treated with RT+CpG+OX40 and the three other groups. Treatment with RT+CpG+OX40 significantly upregulated expression of chemokine genes (*Ccl2*, *Ccl3*, and *Ccl4*) over PBS and RT alone, suggesting increased immune cell trafficking to the TME (**Figures 4A–D**) (37). RT+CpG+OX40 also significantly increased expression of type-I IFN pathway genes (*Oas2*, *Pd-l1*, and *Ifn-alpha receptor 1*) compared to PBS treated control tumors (**Figures 4E–H**). Acute phase cytokine gene expression (*Il-6*, *Il-1 beta*, and *Il-1 alpha*) was significantly increased, or demonstrated a trend towards increased expression (Tnf-alpha), following RT+CpG+OX40 (**Figures 4I–L**), as was the expression of immune cell adhesion proteins *Vcam-1* and *Icam-1* (**Figures 4M, N**), in comparison to PBS treated control tumors. In addition to these changes related to inflammation and immune cell recruitment to the TME, we also observed that the combination of RT+CpG+OX40 significantly increased expression of *Mhc-I* and G*ranzyme b* compared to treatment with PBS (**Figures 4O, P**). Yet, because these data represent gene expression changes within the TME (and not tumor cells themselves), we cannot be certain which specific cell types within the TME showed expression changes for these genes, but these data do suggest that the combined treatment effects make cells within the TME more susceptible for T cell responses. However, for each of these markers of type I IFN, inflammation, tumor cell immune susceptibility, and immune cell activation, we did not observe any significant differences in gene expression between the groups treated with CpG+OX40 *vs.* RT+CpG+OX40. This suggested that the effects we observed on gene expression with RT+CpG+OX40 on day 14 after RT, while potentially critical to the *in situ* vaccine effect, were not likely to be the primary driver of the RT-dependent changes in the TME that enabled an anti-tumor response to CpG+OX40.

### RT+CpG+OX40 Improves IT Effector to Treg Ratios

**Figure 5:**
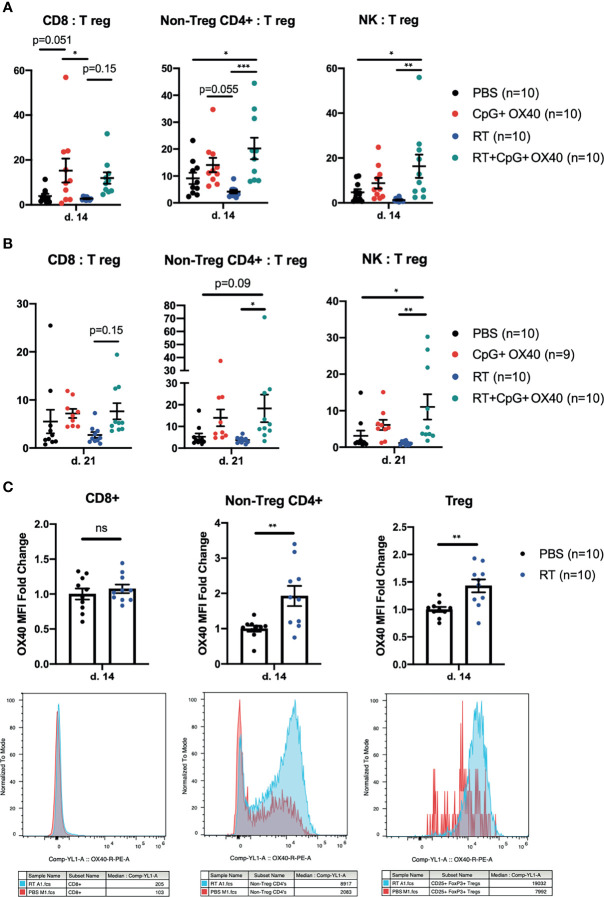
Phenotypic Changes in TIL Following *In Situ* Vaccination. **(A, B)** The combined results from two independent experiments (n=4-5 per group, per experiment) showing the ratios of CD8:Treg, CD4+ non-Treg : Treg, and NK : Treg within the B78 TME **(A)** 14 days and **(B)** 21 days after treatment with PBS (black), CpG+OX40 (red), RT (blue), and RT+CpG+OX40 (teal). **(C)** The combined results from two independent experiments (n=5 per group, per experiment) showing the fold-change in OX40 MFI expression on CD8+, CD4+ non-Treg cells, and Tregs within B78 tumors 14 days after treatment with PBS (black) or RT (blue). Representative histograms for each T cell subpopulation provided below fold-change figures. Average OX40 MFI values in the PBS cohort were calculated for each separate experiment, then used to determine fold change differences for each sample from that particular experiment. Each symbol represents the TIL from one mouse. Flow gating strategy is presented in **Supplementary Figure 4**. P values for effector/Treg ratios were calculated using one-way ANOVA with Tukey’s multiple comparison tests. P values for OX40 expression were calculated using a Mann-Whitney test. *P ≤ 0.05; **P ≤ 0.01; ***P ≤ 0.001; NS, nonsignificant.

Next, we investigated the type of immune cells in the TME following treatment with RT+CpG+OX40. B78 tumors treated with PBS, CpG+OX40, RT, and RT+CpG+OX40 were harvested on days 14 or 21 after treatment initiation, disaggregated, and analyzed by flow cytometry. On day 14, RT+CpG+OX40 significantly improved the non-Treg CD4+:Treg and NK : Treg ratios compared to PBS and RT alone but did not change these ratios compared to CpG+OX40 (**Figure 5A**); furthermore, RT+CpG+OX40 had no effect on the CD8:Treg ratio compared to CpG+OX40 (**Figure 5A**). Compared to PBS and RT, both CpG+OX40 and RT+CpG+OX40 reduced the frequency of Tregs in the B78 TME but did not significantly alter the relative frequency of CD8+ and CD4+ non-Tregs (**Supplementary Figure 5**). Therefore, we found the decrease in tumor Tregs to be the driving factor in the improved effector to Treg ratios following treatment with CpG+OX40 and RT+CpG+OX40.

These trends of effector to Treg ratios measured on day 14 after treatment remained similar on day 21 post treatment (**Figure 5B**). RT+CpG+OX40 significantly increased the non-Treg CD4+:Treg ratio compared to treatment with RT alone and showed a trend (p=0.0851) toward an increased ratio when compared to PBS treated tumors. RT+CpG+OX40 maintained an improved CD8:Treg ratio compared to RT alone, but the increase was not significant (p=0.146) (**Figure 5B**). RT+CpG+OX40 maintained a significantly increased NK : Treg ratio compared to PBS and RT treatment cohorts. RT may modestly and favorably impact this tumor infiltrating lymphocyte composition although not at a level that reaches significance when comparing RT+CpG+OX40 to CpG+OX40. Given the clear effect of RT on anti-tumor response to CpG+OX40, it is not likely that these subtle effects of RT alone give rise to the observed role of RT in facilitating a response to CpG+OX40 in immunologically “cold” tumors.

### RT Stimulates OX40 Expression on Tumor Infiltrating CD4+ T Cells

Others have shown that RT can induce OX40 expression on tumor infiltrating T cells (38). Since the administration of RT prior to CpG+OX40 significantly improved local tumor control in multiple tumor models, we investigated whether OX40 expression changes were occurring in lymphocytes from the immunologically “cold” B78 TME following RT. Mice bearing B78 tumors were randomized and treated with PBS or RT, and tumors were harvested on post-treatment day 14 for flow cytometry analysis. RT significantly increased OX40 expression on tumor infiltrating CD4+ non-Tregs and CD4+ Tregs (**Figure 5C**). There was no difference in OX40 expression on CD8+ TILs 14 days after RT (**Figure 5C**). OX40 expression has previously been shown to be induced on T cell immune populations after T cell receptor engagement and in the presence of proinflammatory cytokines like IL-12 (39). Therefore, these data suggest that RT increases antigen presentation to the CD4+ non-Treg and Treg populations, resulting in increased OX40 expression (13).

### RT Increases CD4+ and CD8+ T Cell Activation in the TDLN and Spleen When Combined With CpG+OX40

**Figure 6:**
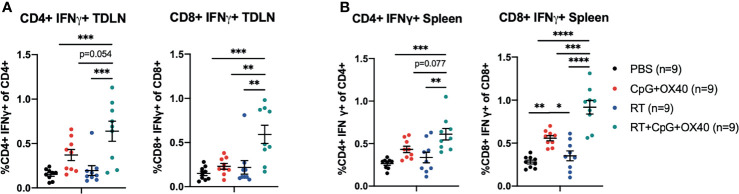
RT+CpG+OX40 Increases CD4+ and CD8+ T Cell Activation in the TDLN and Spleen. The combined results from two independent experiments (n=4-5 per group, per experiment) showing the percent of CD4+ and CD8+ T cells that were positive for IFNγ, without *ex vivo* stimulation, in the TDLNs **(A)** and spleens **(B)** of mice treated with PBS (black), CpG+OX40 (red), RT (blue), and RT+CpG+OX40 (teal). Each symbol represents the immune cell double positive percent from one mouse. Flow gating strategy for these analyses is presented in **Supplementary Figure 5**. P values were calculated using one-way ANOVA with Tukey’s multiple comparison tests. *P ≤ 0.05; **P ≤ 0.01; ***P ≤ 0.001; ****P ≤ 0.0001.

Given that RT may be increasing antigen presentation to CD4+ T cells, resulting in increased OX40 expression, we hypothesized that RT could be functioning to improve T cell priming for subsequent activation with CpG+OX40. To investigate this question, B78 tumor bearing mice were randomized and treated with PBS, CpG+OX40, RT, and RT+CpG+OX40. On day 12 after RT, TDLNs and spleens were harvested for flow cytometry and analyzed for IFNγ expression without additional *ex vivo* stimulation. In the TDLN, RT+CpG+OX40 significantly increased the percent of CD4+ T cells that were IFNγ+ compared to PBS or RT treated mice (**Figure 6A**). CD4+ T cells in the TDLN from mice treated with RT+CpG+OX40 demonstrated a trend towards increased levels of activation compared to CD4+ T cells in the TDLN from mice treated with CpG+OX40 (p=0.054). A significantly increased percent of CD8+ T cells in the TDLN from mice treated with RT+CpG+OX40 were positive for IFNγ compared to mice treated with PBS, RT, and CpG+OX40 (**Figure 6A**).

Similar trends of T cell activation following RT+CpG+OX40 were measured in the spleen, where RT+CpG+OX40 significantly increased the percent of CD4+ T cells that were IFNγ+ compared to treatment with PBS and RT alone (**Figure 6B**). There was a trend towards increased levels of IFNγ+ CD4+ T cells in the spleen following RT+CpG+OX40 compared to CpG+OX40 alone (p=0.077). RT+CpG+OX40 also significantly elevated levels of CD8+ T cell activation in the spleen compared to PBS, RT, and CpG+OX40, as measured by the percent of CD8+ T cells that were IFNγ+ (**Figure 6B**).

## Discussion

We confirmed that CpG and OX40 work together to cure mice bearing A20 tumors, reproducing the results of others with this same *in situ* vaccine regimen (9–11). However, when we tested this same regimen in immunologically “cold” B78 melanoma or 4T1 breast tumor models, we failed to measure any significant anti-tumor effect. Our data suggest that the difference in response to CpG+OX40 between the A20 and B78 models may be due, at least in part, to different effects of CpG on the expression of OX40 on T cells infiltrating these tumors. CpG, a TLR9 agonist currently being tested in clinical trials either alone or in combination with other therapies, can increase Type I IFN production, promote increased antigen presentation by APCs, and result in the generation of adaptive immune responses (12, 40). Sagiv-Barfi et al. published that IT CpG can increase OX40 expression on tumor CD4+ effector cells, providing mechanistic rationale for combining CpG and OX40 (11). Our gene expression data suggests that CpG functions to increase Type I IFN in the B78 TME (**Figures 4E–H**). However, CpG fails to increase OX40 expression on CD4+ non-Treg and CD8+ cells in the B78 model, an activity that is observed in the A20 model, suggesting a failure of adequate T cell priming in the B78 model (**Figures 2A, B**). Additionally, CpG+OX40 fails to increase T cell activation in the TDLN to levels that are presumably required for an effective anti-tumor response (**Figure 6A**). In the B78 model, RT increases OX40 expression on tumor CD4+ cells (**Figure 5C**) and increases T cell activation in the spleen and TDLN when combined with CpG+OX40 (**Figures 6A, B**). OX40-ligand-receptor engagement on CD4+ and CD8+ effector cells promotes T cell activation and survival (13, 15, 16). Since OX40 is transiently expressed on T effector populations after antigen stimulation, our data suggest that RT is assisting the *in situ* vaccine effect of CpG+OX40 by increasing T cell priming in the B78 model (13, 41). We have previously reported RT’s effect on increasing Batf3 dendritic cell frequency, an immune cell subpopulation that has important roles in T cell priming and effector T cell trafficking, in the B78 TME compared to a vehicle control (~2% *vs* 0%, respectively) (42–45). In our studies, subsequent injection of CpG+OX40 after RT appears to be activating primed T cells for immune-mediated tumor cell killing.

Whether the induction of OX40 expression on T cells in the TME induced by CpG in A20 but not in B78 tumors reflects the differences in the cancer cell biology of the A20 and B78 tumor or the differences in the CpG responsiveness of immune cells invading these two tumors requires further study. Though we did not explore the frequency of antigen presenting cells in the TME of B78 and A20 tumors in this manuscript, we have investigated the frequency of dendritic cells and macrophages in the B78 TME for a separate, unrelated manuscript that is in preparation. Based on these data and the published literature, we believe A20 and B78 tumor models have similar, baseline levels of these CpG responsive immune subpopulations (B78 macrophage frequency ~25% of CD45+, B78 DC frequency ~2% of CD45+ *vs* A20 macrophage frequency ~30% of CD45+, A20 DC frequency ~1-2% of CD45+) (46). Even though CpG does not increase OX40 on T cells in the B78 TME in C57BL/6 mice, CpG is active *in vitro* and *in vivo* in C57BL/6 mice (47). This includes *in vivo* activation of IFN-dependent macrophage anti-tumor activity against the B78 related B16 melanoma and IL-12 production from *ex vivo* CpG activated macrophages (47, 48). Furthermore, the combination of CpG and agonist anti-CD40 mAb is able to induce immune-mediated anti-tumor effects against the B78 melanoma in C57BL/6 mice (49) and augment destruction of B78 tumors *in vivo* when combined with an anti-GD2 mAb reactive with B78 (50). These data presented here suggest that CpG is behaving differently in the A20 and B78 models; however, our previous work with CpG indicates that it is contributing to the anti-tumor response we observe with RT+CpG+OX40.

While the stark difference observed here in the potent anti-tumor activity of CpG+OX40 in A20 tumors (~350 mm^3^) compared to the absence of any effect in B78 tumors (~150 mm^3^) might reflect the difference in responsiveness of “hot” *vs.* “cold” tumors, CpG+OX40 may induce some anti-tumor effects against much smaller B78 tumors. We have previously shown that differences in tumor size (22), including for the B78 tumor (23, 32), can influence response to other *in situ* vaccine therapies in preclinical models. In preliminary data that will be submitted in a separate report, we have explored how tumor size can substantially influence the outcome of both A20 and B78 tumors to this same CpG+OX40 *in situ* vaccine regimen, as well as potential mechanisms underlying these size-dependent differences in response.

The measured local anti-tumor benefit of adding RT to CpG+OX40 in the “cold” B78 and 4T1 models is clear. However, we did not find any significant differences between RT+CpG+OX40 and CpG+OX40 in our effector to Treg ratios or gene expression experiments. The CpG+OX40 combination is improving the immune effector to Treg ratios in the TME and is favorably changing the gene expression of the TME, but the *in situ* vaccine is not consistently causing an anti-tumor response in the B78 or 4T1 models. This is likely a result of the *in situ* vaccine failing to adequately activate T cells. RT has been shown to modify the TME of a “cold” tumor, making tumors more immunogenic and susceptible to immune cell killing (2, 25, 51). In the B78 model, RT increases OX40 expression on CD4+ TILs (both Tregs and non-Tregs) in the TME. We explored the potential additive effect of RT combined with CpG on OX40 expression in the B78 model but failed to see a further increase in OX40 expression on CD4+ TILs when RT is combined with CpG (data not shown). Here, we report RT increases CD4+ and CD8+ T cell activation in the TDLN and spleen when combined with CpG+OX40. Taken together, our data suggests RT is improving the in-situ vaccine CpG+OX40 by altering the number of tumor specific T cells in the TME rather than significantly altering the T cell frequency. While previous studies have shown both CD4+ and CD8+ T cells are important for the adaptive anti-tumor response following CpG+OX40 (11), ongoing research aims to understand the relative importance of these populations in the RT+CpG+OX40 regimen.

Beyond its effects on immune cell modulation, we have previously shown that RT modifies the gene expression of B78 tumors *in vivo*, making the tumor cells more susceptible to immune-mediated killing (24). *Fas*, *Dr5*, *Trail-2*, *Mhc-II*, and C*alreticulin* gene expression all increase in a time-dependent manner following RT, with expression peaking at day 7 after RT (24, 52). These effects of RT on tumor cell immune susceptibility could be contributing to the improved anti-tumor response observed with combined RT+CpG+OX40 therapy. Additionally, RT has known effects on the tumor vasculature, increasing endothelial expression of intracellular adhesion molecule-1 (ICAM-1) and vascular cell adhesion molecule (VCAM) (53). OX40/OX40L interactions are typically considered in the context of T cell activation, however, there are reports of T cell/endothelial cell interactions utilizing this pathway for improved cell adhesion and T cell extravasation (54–56). Though we did not explicitly explore this concept within this manuscript, it is possible RT is affecting OX40L expression on tumor endothelial cells, increasing the frequency of T cell/endothelial cell adhesion interactions, and assisting in T cell extravasation from the vasculature into the TME.

In the 4T1 model, RT+CpG+OX40 significantly improved local tumor control over PBS, CpG+OX40, and RT alone. In this model, however, the local tumor control did not translate to an improvement in overall survival. The lack of an effective systemic anti-tumor response in this model could be a result of the immune suppressive microenvironment at distant, untreated metastatic lesions, which is inhibiting propagation of an adequately primed adaptive response. Delivering RT to these metastatic sites of disease could render them susceptible to the *in situ* vaccine immune response generated at the primary tumor. However, delivering external beam RT to all sites of disease remains clinically challenging and is not feasible for radiographically occult tumor sites. Targeted radionuclide therapy (TRT) is a growing class of therapeutics that utilize a tumor-selective vector to deliver radionuclides to tumor sites following intravenous injection. As the TRT agents are selectively taken up by tumors, they deliver their radioactive payload to all tumor sites as the radionuclide decays in the TME. Low-dose TRT can alter the gene expression in the TME and augment response to immune checkpoint blockade (6, 34). In this way, TRT may be a helpful addition to *in situ* vaccine approaches such as RT+CpG+OX40, assisting the propagation of the *in situ* vaccine-activated adaptive immune response at distant sites of immunologically “cold” metastases. The addition of immune checkpoint blockade may also improve the systemic response generated by the local *in situ* vaccine RT+CpG+OX40. The addition of anti-CTLA-4 to CpG+OX40 has been shown to improve the abscopal response in a two-tumor A20 model (9) by increasing the number of IFN-gamma producing CD4+ and CD8+ T cells and further decreasing the Treg frequency in the TME.

## Conclusion

The preclinical data presented here demonstrate the requirement of RT for an effective local anti-tumor effect with CpG+OX40 in two immunologically “cold” tumor models. In the B78 model, RT increases OX40 expression on CD4+ cells in the TME, priming an *in situ* vaccine effect with CpG+OX40. RT activates CD4+ and CD8+ T cells in the TDLN and spleen when combined with CpG+OX40 in these immunologically “cold” tumor models. RT+CpG+OX40 significantly improves local tumor control for both B78 and 4T1 tumors and significantly improves survival for B78 tumor-bearing mice. Given the ongoing clinical trial testing CpG+OX40 in patients with advanced or metastatic solid tumors, adding RT to the *in situ* vaccine CpG+OX40 to improve the adaptive anti-tumor immune response in patients warrants further investigation.

## Data Availability Statement

The raw data supporting the conclusions of this article will be made available by the authors, without undue reservation.

## Ethics Statement

The animal study was reviewed and approved by University of Wisconsin Institutional Animal Care and Use Committee.

## Author Contributions

AP and LZ were responsible for experimental design, execution, and analysis of data. AP created final versions of all figures and drafted the manuscript. DS, RB, and IA collected and analyzed experimental data. RB, IA, RP, NT, AE, DS, and AF collected tissue for flow cytometry analysis. PC, RP, and AF designed and tested the antibody panel used for the flow cytometry experiments. AH, AF, and MF assisted in flow cytometry analysis. JB and TL conducted and confirmed statistical analysis of experimental data. JJ, AB, RP, and AE assisted to design, conduct, and analyze qPCR experiments. PS, AR, RP, and ZM contributed to experimental design, thorough edits, and review of the manuscript. AR, JH, and AE contributed to experimental design, data collection, mouse colony maintenance, and literature research. PS, RP, and ZM provided experimental design and review of data. All authors provided thorough review and editing of the manuscript draft. All authors contributed to the article and approved the submitted version.

## Funding

This work was supported by Midwest Athletes Against Childhood Cancer; Stand Up 2 Cancer; the St. Baldrick’s Foundation; the Crawdaddy Foundation; and the University of Wisconsin Carbone Cancer Center. This research was also supported in part by public health service grants TR002373, U54-CA232568, R35-CA197078, 5K08CA241319, 1DP5OD024576, U01-CA233102, P50 DE026787, P01 CA250972 from the National Cancer Institute; the National Institutes of Health and the Department of Health and Human Services. AP was supported by NIH award TL1 TR002375; PC was supported by the NIH-NCI award F30CA228315 and NIH award TL1 TR002375. JJ was supported by the NIH-NCI award F30CA250263 and NIH award TL1 TR002375. AP, PC, and JJ were funded by T32 GM140935. The content is solely the responsibility of the authors and does not necessarily represent the official views of the National Institutes of Health.

## Conflict of Interest

ZM is a member of the scientific advisory board for Archeus Technologies and Seneca Therapeutics and received equity options for these companies. ZM is an inventor on patents or filed patents managed by the Wisconsin Alumni Research Foundation relating to the interaction of targeted radionuclide therapies and immunotherapies, nanoparticles designed to augment the anti-tumor immune response following radiation therapy, and the development of a brachytherapy catheter capable of delivering intra-tumor injectables. PS is an inventor on patents or filed patents managed by the Wisconsin Alumni Research Foundation relating to mAb-related immunotherapies and the interaction of targeted radionuclide therapies and immunotherapies.

The remaining authors declare that the research was conducted in the absence of any commercial or financial relationships that could be construed as a potential conflict of interest.

The reviewer WS declared a shared affiliation with one of the authors, RP, to the handling editor at the time of the review.

## Publisher’s Note

All claims expressed in this article are solely those of the authors and do not necessarily represent those of their affiliated organizations, or those of the publisher, the editors and the reviewers. Any product that may be evaluated in this article, or claim that may be made by its manufacturer, is not guaranteed or endorsed by the publisher.

## Supplementary Material

Supplementary Figure 1
**(A)** Average tumor volume plots (+/- standard error of the mean), and **(B)** overall survival in the B78 model from a single experiment, with 4 mice per group, showing group responses to CpG+4 mg OX40 (blue), CpG+20 mg OX40 (red), CpG+100 mg OX40 (green). **(C)** Individual tumor mouse curves for the mice shown in **(A, B)**. Time-weighted average analysis and log rank tests did not yield any significant differences.

Supplementary Figure 2
**(A)** The ratio of Tregs (CD3+ CD4+ CD25+ FoxP3+) to live cells from two independent experiments in untreated B78 tumors (blue) or untreated A20 tumors (red). Each symbol represents the TILs from one mouse. Gating strategy to quantify Tregs is shown in **Supplementary Figure 3**. P values for TIL frequency were calculated using a Mann-Whitney test. *P ≤ 0.05; **P ≤ 0.01; ***P ≤ 0.001; ****P ≤ 0.0001.

Supplementary Figure 3 The flow cytometry gating strategy used in FlowJo to quantify OX40 MFI for Tregs and CD4+ non-Tregs (**Figures 2A, B**) and tumor Treg frequency (**Supplementary Figure 2A**).

Supplementary Figure 4
**(A)** The flow cytometry gating strategy used in FlowJo to quantify OX40 MFI on CD4+ T cells (Tregs and non-Tregs) and the ratio of effector cells to Treg cells in the TME for **Figures 5A–C**. **(B)** Representative dot plots of CD4 vs CD8 TILs from groups treated with PBS, CpG+OX40, RT, and RT+CpG+OX40.

Supplementary Figure 5 The frequency of Tregs per 100,000 cells in the TME on d. 14 and d. 21 following treatment with PBS (black), CpG+OX40 (red), RT (blue), and RT+CpG+OX40 (teal). Each symbol represents the TILs from one mouse. P values were calculated *via* two-way ANOVA. *P ≤ 0.05; **P ≤ 0.01; ***P ≤ 0.001; ****P ≤ 0.0001.

Supplementary Figure 6 The flow cytometry gating strategy used in FlowJo to quantify the IFNγ expression levels of CD4+ and CD8+ T cells in the TDLNs and spleens of mice treated with PBS, CpG+OX40, RT, and RT+CpG+OX40 for **Figures 6A, B**.
