# Supplementary figures and images for: Radiation Augments the Local Anti-Tumor Effect of In Situ Vaccine With CpG-Oligodeoxynucleotides and Anti-OX40 in Immunologically Cold Tumor Models
Source: Front Immunol. 2021 Nov 15;12:763888. doi: 10.3389/fimmu.2021.763888 (PMC8634717; doi:10.3389/fimmu.2021.763888)

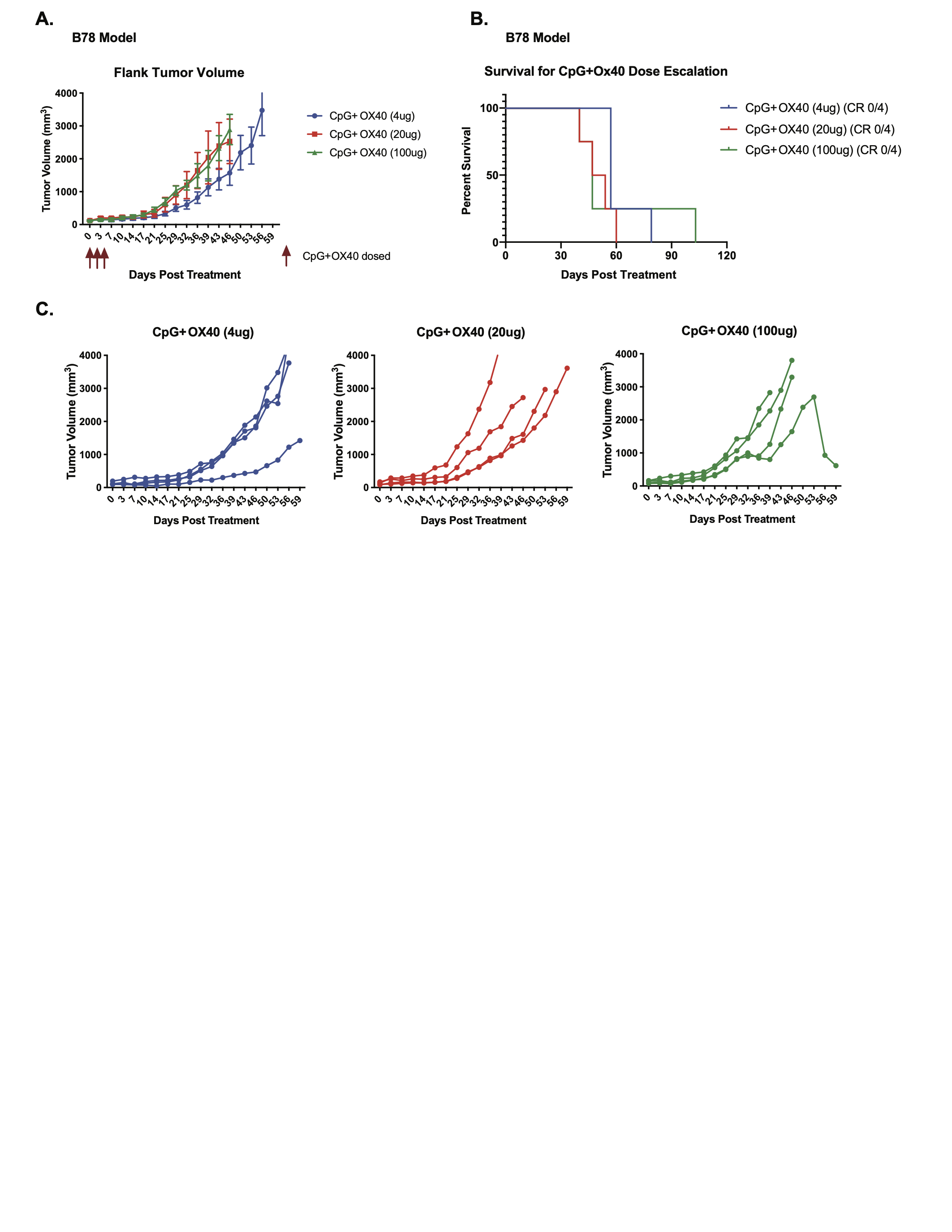

Supplement: Supplementary Figure 1 — (A) Average tumor volume plots (+/- standard error of the mean), and (B) overall survival in the B78 model from a single experiment, with 4 mice per group, showing group responses to CpG+4 mg OX40 (blue), CpG+20 mg OX40 (red), CpG+100 mg OX40 (green). (C) Individual tumor mouse curves for the mice shown in (A, B). Time-weighted average analysis and log rank tests did not yield any significant differences. [file Image_1.tiff]

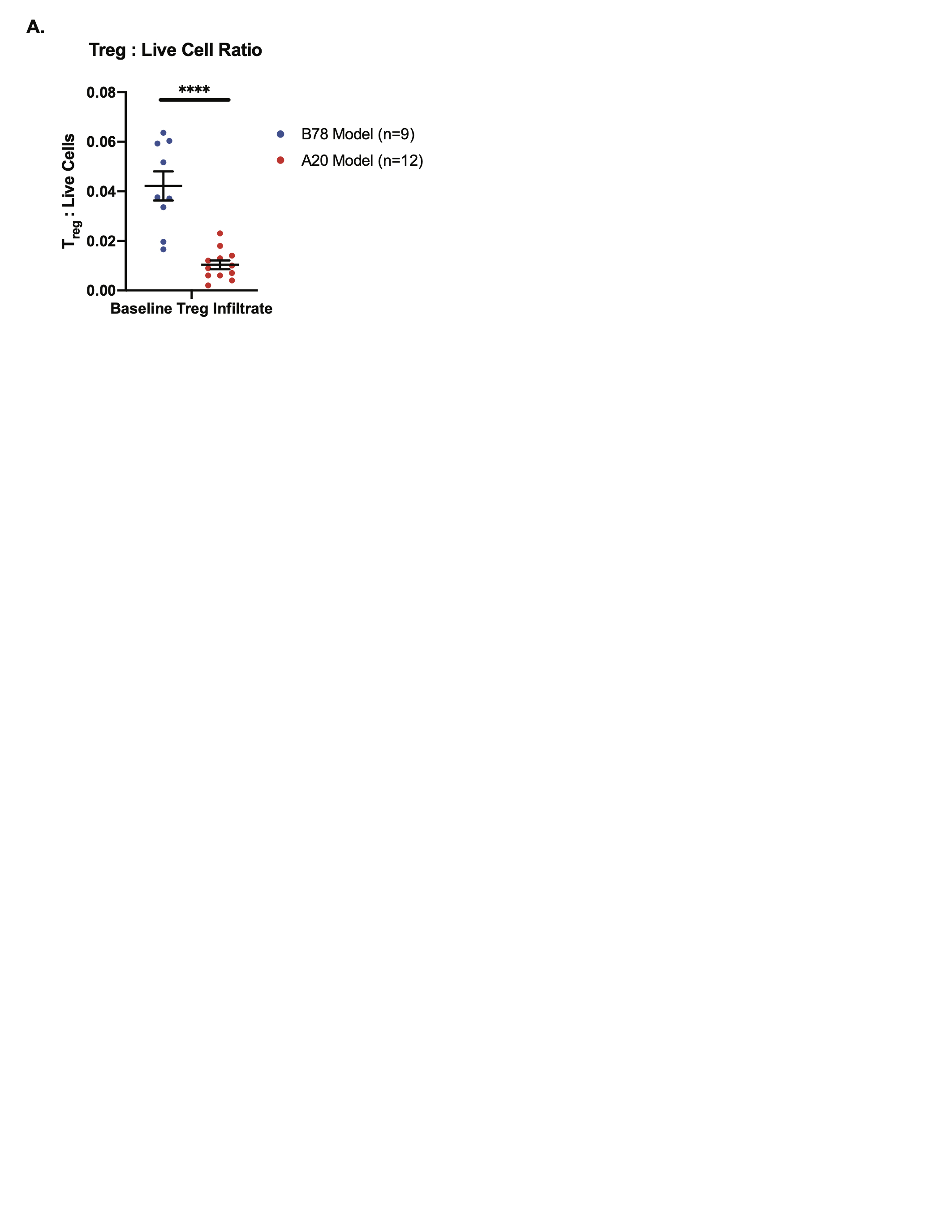

Supplement: Supplementary Figure 2 — (A) The ratio of Tregs (CD3+ CD4+ CD25+ FoxP3+) to live cells from two independent experiments in untreated B78 tumors (blue) or untreated A20 tumors (red). Each symbol represents the TILs from one mouse. Gating strategy to quantify Tregs is shown in Supplementary Figure 3 . P values for TIL frequency were calculated using a Mann-Whitney test. *P ≤ 0.05; **P ≤ 0.01; ***P ≤ 0.001; ****P ≤ 0.0001. [file Image_2.tiff]

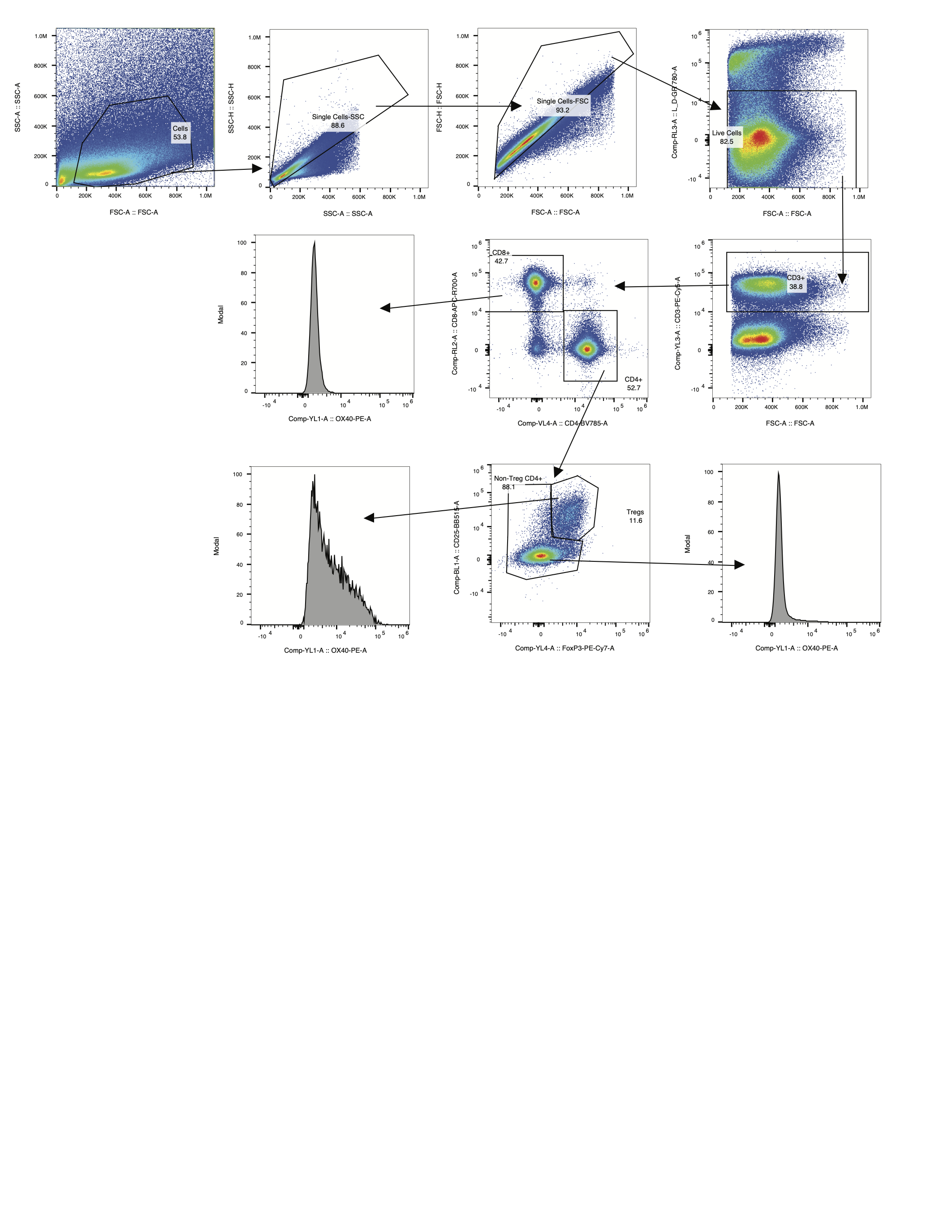

Supplement: Supplementary Figure 3 — The flow cytometry gating strategy used in FlowJo to quantify OX40 MFI for Tregs and CD4+ non-Tregs ( Figures 2A, B ) and tumor Treg frequency ( Supplementary Figure 2A ). [file Image_3.tiff]

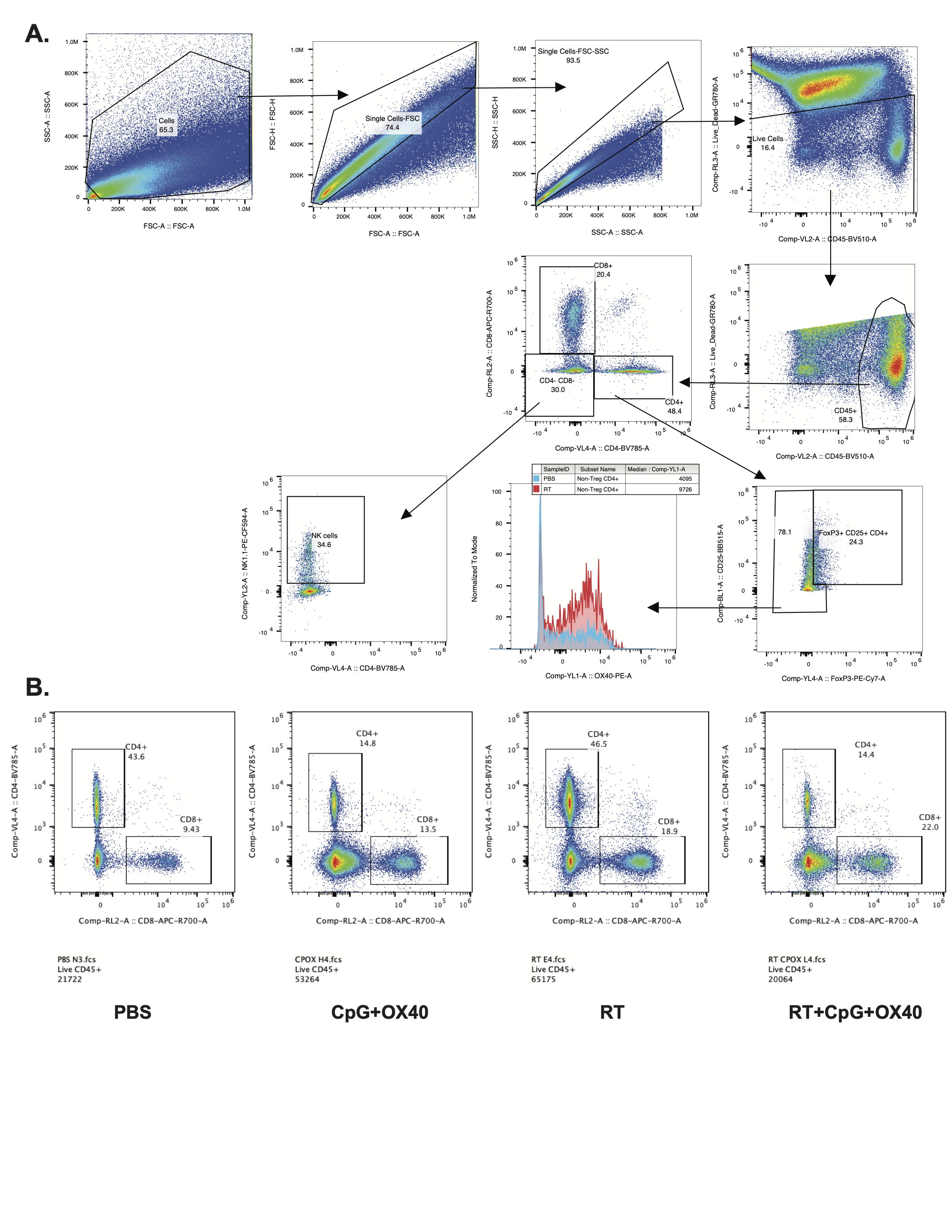

Supplement: Supplementary Figure 4 — (A) The flow cytometry gating strategy used in FlowJo to quantify OX40 MFI on CD4+ T cells (Tregs and non-Tregs) and the ratio of effector cells to Treg cells in the TME for Figures 5A–C . (B) Representative dot plots of CD4 vs CD8 TILs from groups treated with PBS, CpG+OX40, RT, and RT+CpG+OX40. [file Image_4.tiff]

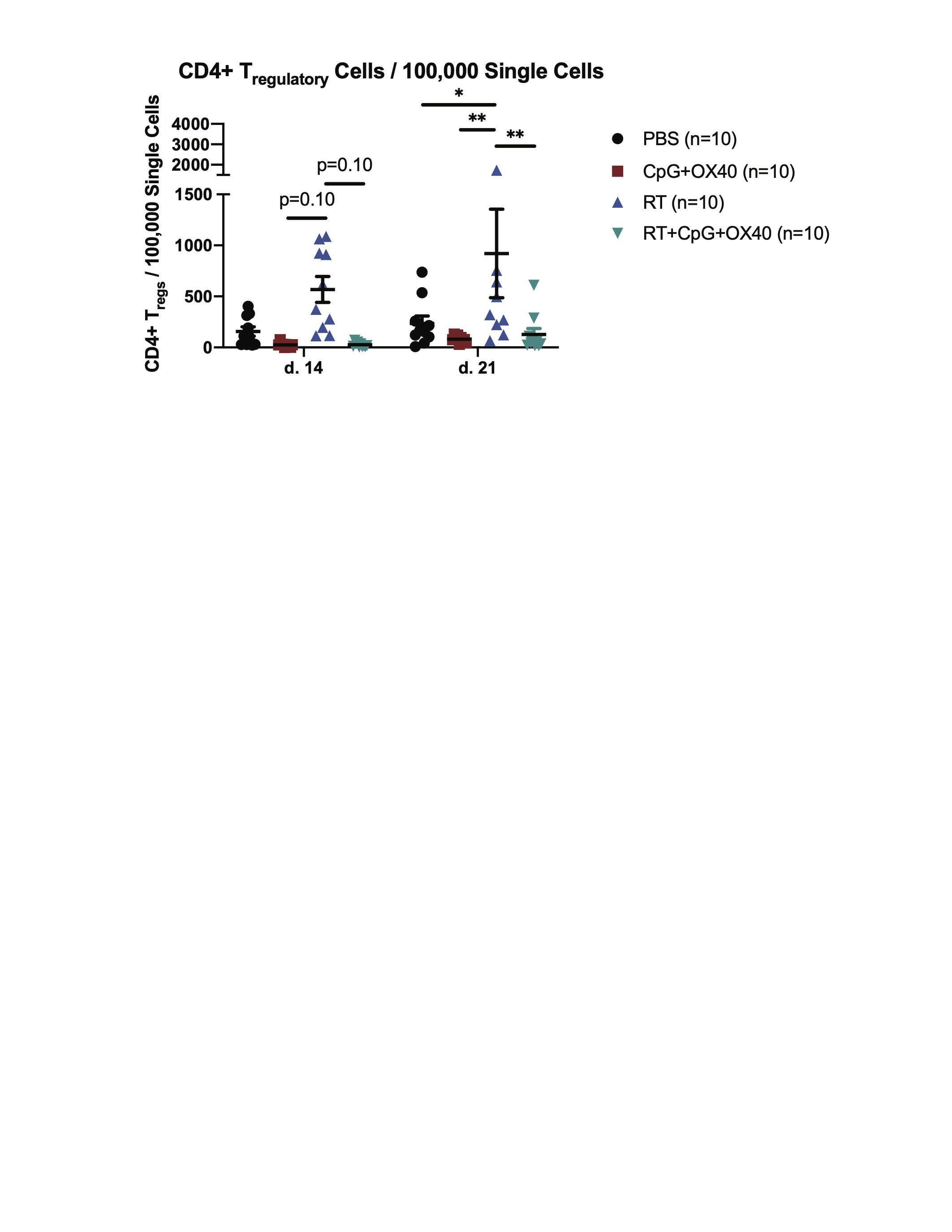

Supplement: Supplementary Figure 5 — The frequency of Tregs per 100,000 cells in the TME on d. 14 and d. 21 following treatment with PBS (black), CpG+OX40 (red), RT (blue), and RT+CpG+OX40 (teal). Each symbol represents the TILs from one mouse. P values were calculated via two-way ANOVA. *P ≤ 0.05; **P ≤ 0.01; ***P ≤ 0.001; ****P ≤ 0.0001. [file Image_5.tiff]

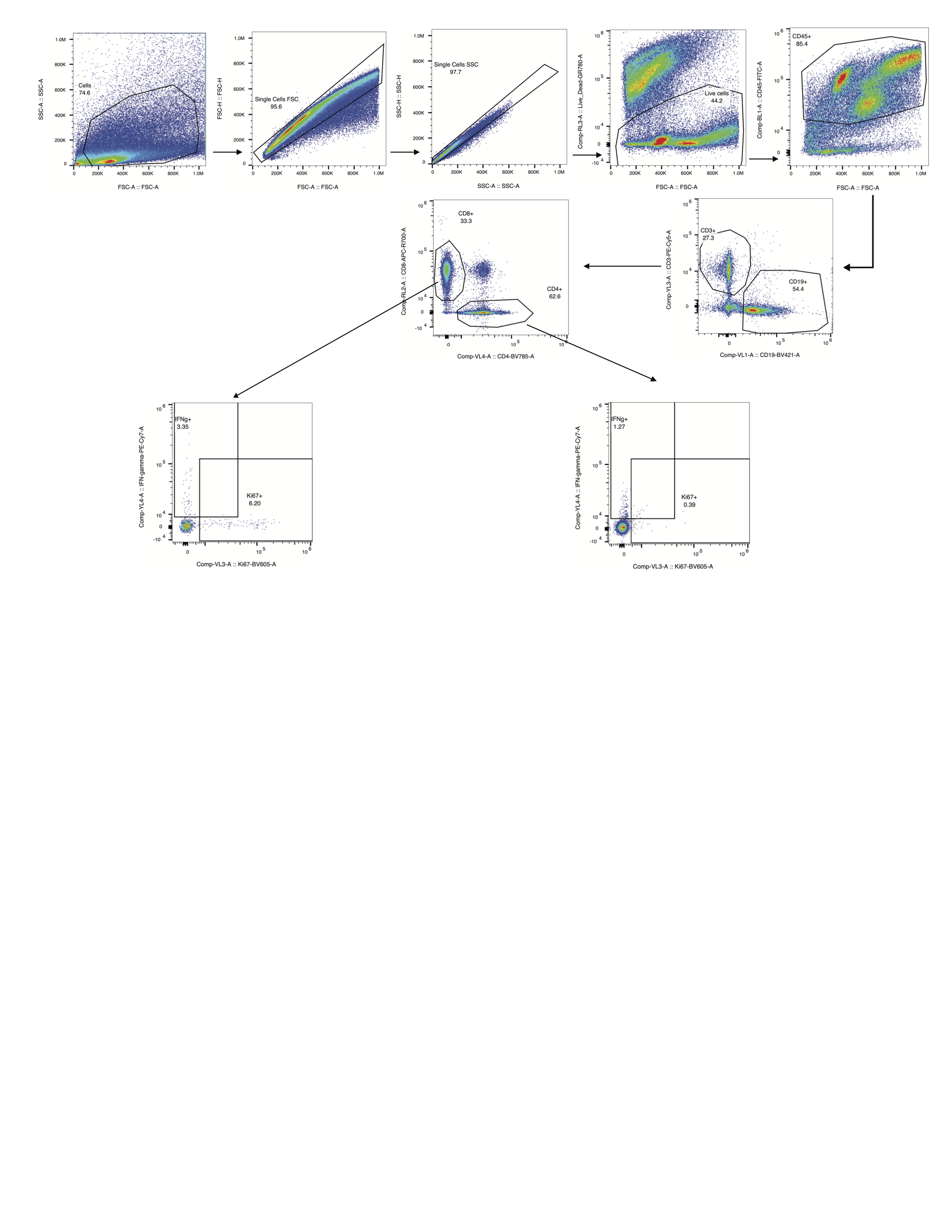

Supplement: Supplementary Figure 6 — The flow cytometry gating strategy used in FlowJo to quantify the IFNγ expression levels of CD4+ and CD8+ T cells in the TDLNs and spleens of mice treated with PBS, CpG+OX40, RT, and RT+CpG+OX40 for Figures 6A, B . [file Image_6.tiff]
